# Physical activity and subsequent risk of kidney, bladder and upper urinary tract cancer in the Japanese population: the Japan Public Health Centre-based Prospective Study

**DOI:** 10.1038/s41416-019-0392-y

**Published:** 2019-02-12

**Authors:** Hikaru Ihira, Norie Sawada, Taiki Yamaji, Atsushi Goto, Taichi Shimazu, Manami Inoue, Motoki Iwasaki, Shoichiro Tsugane, for the Japan Public Health Centre-based Prospective Study Group

**Affiliations:** 1 Epidemiology and Prevention group, Centre for Public Health Sciences, National Cancer Centre, 5-1-1 Tsukiji Chuo-ku, Tokyo, 104-0045 Japan; 2 JPHC Study Group members are listed at, http://epi.ncc.go.jp/en/jphc/781/3838.html

**Keywords:** Cancer epidemiology, Risk factors

## Abstract

**Background:**

Although physical activity has been reported as a protective factor for kidney and bladder cancer in epidemiological studies, it is not clear.

**Methods:**

In a population-based prospective study of 76,795 Japanese aged 45–74 years, participants were evaluated physical activity by self-administered questionnaire. Hazard ratios (HRs) and 95% confidence intervals (CIs) of the risk of urological cancer were calculated by the Cox proportional hazards models.

**Results:**

During the 15.1-year follow-up, 202 kidney, 373 bladder and 83 upper urinary tract cancer cases were identified. Physical activity was not significantly associated with kidney, bladder and upper urinary tract cancer risks, with multivariate HRs in the highest versus lowest group of 1.05 (95% CI: 0.74–1.49), 1.06 (95% CI: 0.81–1.39) and 0.80 (95% CI: 0.48–1.35), respectively.

**Conclusions:**

Physical activity was not associated with the risk of urological cancer in the Japanese population.

## Background

Physical activity is known to reduce risks of colorectal, breast and endometrial cancers according to the reports from World Cancer Research Fund;^1,2^ however, no conclusions have been drawn for kidney and bladder cancers. In Japan, the incidences of kidney and upper urinary tract etc. and bladder cancers were the 9th and 15th most common cancers in 2014, respectively, and the age-standardised rate of these cancers incidences is increasing.^3^ Although recent two meta-analyses found that a higher level of physical activity is associated with decreased risks of kidney^4^ and bladder^5^ cancers, the association remains unclear, especially in the Asian populations.^6,7^ Given that the Asian population has body mass index (BMI) and smoking status different from the Western population and that BMI and smoking are risk factors of kidney and bladder cancers, accumulating the evidence of the association between physical activity and these cancers in the Asian population may be helpful for cancer prevention.

In this longitudinal cohort study, we investigated the association between physical activity and the risk of kidney, bladder and upper urinary tract cancer in the Japanese population.

## Materials and methods

### Study population

The Japan Public Health Center-based prospective study (JPHC study) was initiated in 1990 (Cohort I) and 1993 (Cohort II) and included Japanese residents (*n* = 140,420) from 11 public health centres (PHCs) aged 40–69 years. Details of the study design have been reported previously.^8^ The participants were informed of the study objectives, and those who completed the survey questionnaire were regarded as consenting to participation. The study protocol was approved by the Institutional Review Board of the National Cancer Center, Japan. Here, the results of the 5-year follow-up survey were used as the baseline (starting point) and participants were followed up until 2013. The self-administered questionnaire was used to collect information on the medical history and lifestyle variables, such as physical activity and diet of the participants. Among 121,181 participants after exclusion of 19,239 participants who were ineligible, we enrolled 98,512 participants (46,029 men and 52,483 women) aged 45–74 years who provided valid responses (response rate, 81.3%) to the 5-year follow-up questionnaire.

### Assessment of physical activity

Participants were asked about the time spent per day on three types of physical activity, such as heavy physical work or strenuous exercise, sedentary activity and walking and standing by the self-administered questionnaire (Supplementary Materials). We defined metabolic equivalents (METs) of heavy physical work or strenuous exercise, sedentary activity, walking and standing and other activity as 4.5, 1.5, 2.0 and 0.9, respectively.^9^ We then estimated the METs hours per day (METs/day) by multiplying the asked hours spent and METs at each physical activity per day. After the METs/day were summed up for all the physical activities, participants were stratified by METs/day to their physical activity tertile. Spearman’s rank correlation coefficient for the total METs/day and 24-hour physical activity was 0.46.^10^ To analyse the effect of leisure-time sports or exercise on the cancer incidence, participants were also stratified by the frequency of the leisure-time sports or exercise (<1 day/week, 1–2 days/week and ≥3 days/week).

### Follow-up

Participants were followed up from the starting point to 31 December 2013 except for one PHC area, where the follow-up finished by the end of 2012. Cancer incidence was identified by data linkage with population-based cancer registry with the permission of each local government responsible for the cancer registry and by active patient notification from major local hospitals in the study area. Kidney (C64), bladder (C67) and upper urinary tract cancer (C65, renal pelvis and C66, ureter) were coded using the International Classification of Diseases for Oncology, Third Edition (ICD-O-3).

### Statistical analysis

Of the 98,512 participants who responded to the 5-year follow-up questionnaire, participants with the history of kidney cancer (*n* = 38), bladder cancer (*n* = 57), upper urinary tract cancer (*n* = 5) or cerebrovascular disease (*n* = 577), those who reported daily energy intake in the range of upper or lower 2.5% (*n* = 5939) or those with missing data (*n* = 15,101) were excluded from the analysis. The remaining 76,795 participants, (36,670 men and 40,125 women) were included. Hazard ratios (HRs) and 95% confidence intervals (CIs) were calculated using the Cox proportional hazards models to identify the association between physical activity and leisure-time sports or exercise and cancer incidence with adjustment for potential confounders.

## Results

Participants with a higher level of physical activity had lower BMI, greater total energy intake, performed more leisure-time sports or exercise and had a lower prevalence of diabetes (Supplementary Table S1). During 1,168,317 person-years of follow-up (average follow-up period was 15.1 years) for 76,795 subjects, 202 kidney cancer, 373 bladder cancer and 83 upper urinary tract cancer cases were identified. Physical activity and leisure-time sports or exercise were not significantly associated with kidney, bladder and upper urinary tract cancer risks, with multivariate HRs in the highest versus lowest group of 1.05 (95% CI = 0.74–1.49), 1.06 (95% CI = 0.81–1.39) and 0.80 (95% CI = 0.48–1.35) and 0.87 (95% CI = 0.55–1.38), 0.95 (95% CI = 0.69–1.32) and 0.81 (95% CI = 0.38–1.70), respectively (Table 1). Although we calculated each relative risk for the three components (heavy physical work or strenuous exercise, sedentary activity and walking and standing) of the physical activity, we did not find statistically significant associations between either of the components and the urological cancer risk (data not shown). Furthermore, there was no substantial difference in the results by adding sedentary time for adjustment in analyses (data not shown). No substantial difference was seen in the results on stratification by sex, BMI, smoking status or age and on sensitivity analysis by excluding the participants with kidney, bladder or upper urinary tract cancer incidence within first 5-year follow-up period from starting point (data not shown).

**Table 1:** Hazard ratios for kidney, bladder, and upper urinary tract cancer incidence according to physical activity level and leisure-time sports or exercise

Physical activity level (METs/day)					
	Low (21.6–27.9)	Medium (27.1–33.9)	High (34.3–46.3)	*P* trend	Per 1METs/day increment
Kidney					
Number cases	51	64	87		
Person-years	314,077	381,145	473,095		
Model 1 HRs (95% CI)	1.00	0.91 (0.63–1.31)	1.01 (0.71–1.43)	0.89	1.01 (0.85–1.21)
Model 2 HRs (95% CI)	1.00	0.92 (0.63–1.33)	1.05 (0.74–1.49)	0.71	1.03 (0.87–1.23)
Bladder					
Number cases	87	128	158		
Person-years	314,077	381,145	473,095		
Model 1 HRs (95% CI)	1.00	1.04 (0.79–1.36)	1.05 (0.81–1.37)	0.73	1.02 (0.90–1.17)
Model 2 HRs (95% CI)	1.00	1.05 (0.80–1.39)	1.06 (0.81–1.39)	0.69	1.03 (0.90–1.17)
Upper urinary tract					
Number cases	26	24	33		
Person-years	314,077	381,145	473,095		
Model 1 HRs (95% CI)	1.00	0.71 (0.40–1.24)	0.78 (0.46–1.31)	0.39	0.89 (0.68–1.16)
Model 2 HRs (95% CI)	1.00	0.71 (0.41–1.25)	0.80 (0.48–1.35)	0.46	0.90 (0.69–1.18)

Leisure-time sports or exercise					
	<1 day/week	1–2 days/week	≥3 days/week	*P* trend	
Kidney					
Number cases	158	23	21		
Person-years	918,851	125,334	124,132		
Model 1 HRs (95% CI)	1.00	1.04 (0.67–1.61)	0.90 (0.57–1.43)	0.74	
Model 3 HRs (95% CI)	1.00	1.00 (0.64–1.56)	0.87 (0.55–1.38)	0.59	
Bladder					
Number cases	293	36	44		
Person-years	918,851	125,334	124,132		
Model 1 HRs (95% CI)	1.00	0.90 (0.63-1.27)	0.91 (0.66–1.26)	0.49	
Model 3 HRs (95% CI)	1.00	0.89 (0.62-1.27)	0.95 (0.69–1.32)	0.64	
Upper urinary tract					
Number cases	66	9	8		
Person-years	918,851	125,334	124,132		
Model 1 HRs (95% CI)	1.00	1.00 (0.50–2.02)	0.83 (0.40–1.74)	0.66	
Model 3 HRs (95% CI)	1.00	0.98 (0.48–1.97)	0.81 (0.38–1.70)	0.60	

*HRs* hazard ratios, *CI* confidence interval, *METs* metabolic equivalents

Model 1: adjusted for age (5-year categories), sex (male or female), and area (10 public health center areas)

Model 2: adjusted for total energy intake (quintile), coffee (almost none, 1–4 times/week, 1–2 cups/day or ≥3 cups/day), history of diabetes (no, yes), smoking status (never, former, <20 or ≥20 pack-years), alcohol intake status (non - or occasional drinkers, 1–150 g/week or ≥150 g/week), body mass index (<18.5, 18.5–24.9, 25–29 or ≥30), and leisure-time sports or exercise (<1, 1–2 or ≥3 days/week) in addition to adjustment in Model 1

Model 3: adjusted for total energy intake (quintile), coffee (almost none, 1–4 times/week, 1–2cups/day or ≥3cups/day), history of diabetes (no, yes), smoking status (never, former, <20 or ≥20 pack-years), alcohol intake status (non - or occasional drinkers, 1–150 g/week or ≥150 g/week), and body mass index (<18.5, 18.5–24.9, 25–29 or ≥30) in addition to adjustment in Model 1

## Discussion

Physical activity was not significantly associated with the risk of kidney, bladder or upper urinary tract cancer in the Japanese population. The results on the null association between physical activity and kidney or bladder cancer risk are consistent with the previous studies in Asian populations.^6,7^

A recent meta-analysis found that there is an inverse association between physical activity and kidney^4^ and bladder cancers.^5^ The discrepancy of present results may be partly explained by the difference of the BMI and smoking status in the Asian and Western populations. The World Health Organization (WHO) reported that the prevalence of obesity (BMI ≥ 30) in Japan was approximately 2% in men and 3% in women, while it was approximately 19.9% in men and 24.9% in women in the USA.^11^ The BMI in most European countries was also higher than those in the Japanese population. Given that physical activity contributes to the control of body fat and lower prevalence of overweight and obesity in Japan, physical activity may be less effective for cancer prevention in the Japanese population. Additionally, WHO reported that the prevalence of smoker was 43.3% in Japanese men and 27.5% in USA men.^12^ Given the relatively high prevalence of current smoking in Japanese men and the known effect of smoking as risk factor of kidney and bladder cancer, smoking might have cancelled out the effects of physical activity. To our knowledge, this is the first prospective study reporting a null association between physical activity and upper urinary tract cancer risk, however, the results should be confirmed in a large population by other investigators.

We observed slightly decreased risks for kidney and bladder cancers on individuals spending more time on leisure-time sports or exercise than those spending more time on total physical activity, although the association was not statistically significant. These results are in accordance with those of two studies showing that recreational physical activity may be more effective than work-related activity for the prevention of kidney^4^ and bladder^5^ cancer, although the potential reasons are not clear. The Japanese official physical activity guideline for health promotion recommends people of all ages to engage in exercise for more than 30 min at least 2 days per week.^13^ We speculate that participants in the “ ≥3 days/week” category meet the recommendation of the guideline, assuming exercise times of at least 30 min. Although we cannot directly apply our exposure to the recommendation of the guideline because we did not have information on the durations of the leisure-time sports or exercise, we did not observe any protective effects against urological cancer even in those in the “ ≥3 days/week” category.

The study strengths include its prospective design, large sample size, high survey response rate (81.3%), and low rate of loss of follow-up (0.3%). In contrast, the study limitation includes the small number of cases of kidney and upper urinary tract cancers in our Japanese population despite the relatively large sample size and long follow-up periods. In addition, we did not have information on the durations of the leisure-time sports or exercise. In conclusion, physical activity was not associated with the risk of urological cancer such as kidney, bladder and upper urinary tract cancer in the Japanese population.

## Supplementary information

Supplementary Materials

Supplementary Materials

## Data Availability

For information on how to submit an application for gaining access to JPHC data and/or biospecimens, please follow the instructions at http://epincc.go.jp/en/jphc/805/8155.html.
