# Supplementary material for: Physical activity and subsequent risk of kidney, bladder and upper urinary tract cancer in the Japanese population: the Japan Public Health Centre-based Prospective Study
Source: Br J Cancer. 2019 Feb 12;120(5):571–4. doi: 10.1038/s41416-019-0392-y (PMC6461777; doi:10.1038/s41416-019-0392-y)
Supplement: Supplementary file 1 — Supplementary Materials [file 41416_2019_392_MOESM1_ESM.docx]

Supplementary Materials

Question related to physical activity in the self-administered questionnaire at 5-year

follow-up survey of the Japan Public Health Center-based prospective study:

“How long do you usually spend on the following three types of physical activities

including work time?”

| Heavy physical work or strenuous exercise | none | <1 hour | ≥1 hour |
| --- | --- | --- | --- |
| Sedentary activity | <3 hour | From 3 to <8 hour | ≥8 hour |
| Walking and standing | <1 hour | From 1 to <3 hour | ≥3 hour |
